# Fat, Sugar, Whole Grains and Heart Disease: 50 Years of Confusion

**DOI:** 10.3390/nu10010039

**Published:** 2018-01-04

**Authors:** Norman J. Temple

**Affiliations:** Centre for Science, Athabasca University, Athabasca, AB T9S 3A3, Canada; normant@athabascau.ca; Tel.: +1-780-450-0167; Fax: +1-780-675-6186

**Keywords:** coronary heart disease, saturated fat, sugar, whole grains, cereal fiber

## Abstract

During the 1970s some investigators proposed that refined carbohydrates, especially sugar and a low intake of dietary fiber, were major factors in coronary heart disease (CHD). This suggestion was eclipsed by the belief that an excess intake of saturated fatty acids (SFA) was the key dietary factor, a view that prevailed from roughly 1974 to 2014. Findings that have accumulated since 1990 inform us that the role of SFA in the causation of CHD has been much exaggerated. A switch from SFA to refined carbohydrates does not lower the ratio of total cholesterol to HDL-cholesterol in the blood and therefore does not prevent CHD. A reduced intake of SFA combined with an increased intake of polyunsaturated fatty acids lowers the ratio of total cholesterol to HDL-cholesterol; this may reduce the risk of CHD. The evidence linking carbohydrate-rich foods with CHD has been steadily strengthening. Refined carbohydrates, especially sugar-sweetened beverages, increase the risk of CHD. Conversely, whole grains and cereal fiber are protective. An extra one or 2 servings per day of these foods increases or decreases risk by approximately 10% to 20%.

## 1. Introduction

During the 1970s various investigators proposed that the epidemic of coronary heart disease (CHD) is caused, at least in part, by refined carbohydrates. Particular attention focused on sugar and a low intake of dietary fiber. This hypothesis never gained widespread support (apart from a minor role for fiber). At the same time it became widely accepted that the dominant dietary factor involved in CHD was an excessive intake of saturated fatty acids (SFA). This viewpoint became the accepted orthodoxy for four decades (from roughly 1974 to 2014). The pendulum has now swung in the opposite direction: SFA is now believed to play a much smaller role in CHD than was previously believed while that of sugar, whole grains, and cereal fiber have moved in the opposite direction.

This paper looks at the changing viewpoints of the roles played by SFA, sugar, whole grains, and cereal fiber in CHD over the period from the late 1960s to the present.

## 2. Refined Carbohydrates, Sugar, Whole Grains, Dietary Fiber and CHD: The Early Years

In the late 1960s and through the 1970s several medical investigators, all from southern England, proposed that CHD was strongly related to refined carbohydrates, sugar, and a low intake of dietary fiber. The main supporting evidence was based on international comparisons of diet and incidence of CHD.

Yudkin argued that sugar was implicated in several diseases, most notably CHD [1,2]. However, the supporting evidence was weak. First, there was no established mechanism by which sugar might cause CHD. In particular, sugar has only a weak effect on the blood cholesterol level (i.e., total cholesterol, TC). An additional problem with the evidence was that cohort studies published at that time failed to find a clear association between intake of sugar and risk of CHD. Because of the weakness of the evidence, the hypothesis never gained widespread acceptance. One factor, though probably a minor one, was that the sugar industry in the USA paid researchers to publish papers that emphasized the role of SFA in the causation of CHD while minimizing the role of sugar [3]. This scandal only came to light in 2016.

While Yudkin focused narrowly on the role of sugar in CHD and other diseases, Cleave spread the blame more widely on refined carbohydrates (i.e., sugar and products of refined cereals such as white bread) [4,5]. He blamed these foods for not only CHD but also for a host of diseases now known as chronic diseases of lifestyle or non-communicable diseases.

Refined cereals have a much lower content of dietary fiber and of numerous micronutrients compared with unprocessed cereals. White sugar, of course, is totally lacking in fiber and all micronutrients. Most attention has focused on fiber. Burkitt and Trowell were instrumental in formulating the hypothesis that a low intake of fiber plays a central role in several chronic diseases of lifestyle including CHD [6,7]. Burkitt is particularly well known for his discovery of Burkitt’s lymphoma and for his hypothesis that a low intake of fiber plays a major role in the causation of cancer of the colon. Burkitt and Trowell were not the first people to suggest that fiber may help prevent disease. For example, in 1951 Walker, working in South Africa, suggested that fiber may be protective against CHD [8].

One result of this interest in fiber was the discovery that fiber helps lower the blood TC level. However, this property is not shared by all types of fiber: viscous fiber, which is found in oats, beans, and fruit, lowers the TC, whereas insoluble fiber, the type found in wholewheat, is ineffective [9]. While these findings are valuable with respect to the prevention of CHD, they provide no support for the hypothesis that sugar and refined cereals are linked to CHD.

In summary, only weak supporting evidence was available in the 1970s for the hypothesis that refined carbohydrates, sugar, and a low intake of dietary fiber play an important role in the causation of CHD.

## 3. The Age of Saturated Fat

At around the same time that the above debate was taking place, other investigators were studying the relationship between dietary fats and CHD.

Dietary fats consist mostly of triglycerides. These are formed by fatty acids attached to glycerol. The fatty acids have much chemical variation. SFA are fully saturated (i.e., they have no double bonds) and mostly have a chain length of 12 to 18 carbons. Major sources are foods obtained from animals, such as meat and cheese, and tropical oils. Monounsaturated fatty acids have one double bond. The most common type is oleic acid, which has 18 carbons. Rich sources of monounsaturated fatty acids include red meat, nuts, olive oil, and canola oil. Polyunsaturated fatty acids (PUFA) have two or more double bonds. Depending on the location of the first double bond they are classified as either *n*-6 (omega-6) or *n*-3 (omega-3). The predominant *n*-6 PUFA is linoleic acid which has 18 carbons and two double bonds. Vegetable oils such as corn oil, sunflower oil, and soybean oil are especially rich sources. The major type of *n*-3 PUFA is alpha-linolenic acid which has 18 carbons and three double bonds. It is found in vegetable oils including flaxseed oil (a rich source), soybean oil, and to a lesser extent canola oil. Smaller amounts of *n*-3 PUFA occur in seafood as very long-chain fatty acids (20 or 22 carbons) with 5 or more double bonds. Rich sources include fatty fish such as herring, salmon, mackerel, and sardines. All double bonds in PUFA are normally in the cis configuration, but in trans fatty acids one or more double bonds are in the trans configuration.

The Framingham study, a long-term cohort study, was crucial as it demonstrated that the blood TC level is a major risk factor for CHD [10]. It was also established that an increased intake of SFA (and also dietary cholesterol, though to a much lesser extent) increases the blood TC level [11,12]. These findings suggested that SFA is a causative factor of CHD. Support for the hypothesis also came from the Seven Countries study which was carried out in 15 areas in seven European countries. The findings showed a strong correlation between intake of SFA (as a percentage of energy) and mortality from CHD [13].

In contrast to SFA, polyunsaturated fatty acids (PUFA) lower the TC [11,12]. These findings led to the conclusion that substituting PUFA for SFA should help prevent the disease. This possibility was tested in several major randomized controlled trials (RCTs). The findings from these trials have been generally interpreted as indicating that the risk of CHD can be reduced by partially replacing SFA with PUFA [14].

The above evidence, taken as a whole, led to the conclusion that an excess intake of SFA is a major cause of CHD. This verdict became widely accepted after about 1974. However, there was never universal support for the hypothesis. Indeed, several investigators argued that the hypothesis was not consistent with the evidence [15]). For example, Temple [16] argued that the epidemiological evidence supported refined carbohydrates rather than SFA as playing the dominant role in CHD.

## 4. The Pendulum Swings: The SFA-CHD Hypothesis Starts to Unravel

Serious flaws with the SFA-CHD hypothesis have steadily emerged. Many cohort studies were published after 1990 that have provided a wealth of information on the relationship between diet and risk of CHD e.g., [17,18,19,20,21]. Meta-analyses of cohort studies have clearly shown that intake of SFA has only a weak, non-significant association with risk of CHD (risk ratio (RR): 1.06 (95% confidence interval (CI) 0.95–1.17) for total CHD; 1.15 (0.97–1.36) for CHD mortality) when comparing extreme quintiles) [22]. The possibility must be considered that due to methodological errors the true association is much larger than is indicated by cohort studies. This is very unlikely as demonstrated by the fact that cohort studies have reported that several other components of the diet have much stronger, significant associations with risk of CHD. For example, meta-analyses of cohort studies have reported a significant positive association between trans fats and risk of CHD [16] and significant negative associations for fish [23], cereal fiber [24], and fruit and vegetables [25]. A comparison of extreme quintiles indicates that each of these components of the diet raise or lower the risk of CHD by approximately 15–20%, whereas a relatively high intake of SFA causes a smaller impact on risk of CHD. These comparisons demonstrate, first, that when cohort studies report a weak association between SFA and risk of CHD, this cannot be dismissed as methodological error, and, second, that SFA has a much weaker association with CHD than does several other components of the diet.

A few years after the SFA-CHD hypothesis had been widely accepted, it was realized that the relationship between SFA, blood lipids, and risk of CHD was much more complex than had been generally believed. By the late 1970s evidence emerged that LDL-cholesterol is directly related to risk of CHD whereas HDL-cholesterol has an inverse association [26]). The strongest indicator of risk is seen for the ratio of TC to HDL-cholesterol [27]. Partially replacing dietary SFA with PUFA lowers the LDL-cholesterol but without lowering the HDL-cholesterol. As a result the ratio of TC to HDL-cholesterol is decreased which should result in a reduction in risk of CHD. Supporting evidence has come from cohort studies which reveal that a 5% lower intake of SFA combined with a 5% higher intake of linoleic acid (the predominant PUFA) is associated with a lower risk of CHD (RR: 0.91, CI 0.87–0.96, for total CHD; 0.87, CI 0.82–0.94, for CHD mortality) [28]. This has been tested in several RCTs in which dietary SFA was partially replaced with PUFA. As stated earlier the findings from these RCTs have been generally interpreted as indicating that the risk of CHD is reduced by this dietary manipulation. This interpretation of the various lines of evidence concerning the relationship between dietary intake of SFA and PUFA, blood lipids, and risk of CHD is now supported by a majority of authoritative opinion [29,30,31,32].

However, some controversy has emerged in recent years regarding the interpretation of RCTs that aimed to prevent CHD by lowering the TC. Hamley [33] argued that replacing SFA with PUFA does not lower the risk of CHD. He argued that several RCTs have been misinterpreted due to the inclusion of inadequately controlled trials. Ramsden et al. [34] came to the same conclusion. The claim that CHD can be prevented by replacing SFA with PUFA so as to lower the ratio of TC to HDL-cholesterol is therefore still open to debate. These recent re-analyses of RCTs casts further doubt on the SFA-CHD hypothesis.

The SFA-CHD hypothesis became widely accepted after the evidence reached a critical weight. This has now happened in reverse: the weight of evidence now indicates that SFA play only a minor role in CHD [35].

## 5. Back to the Future: The Re-Emergence of the Hypothesis Linking Refined Carbohydrates, Sugar, and Dietary Fiber with CHD

At the same time as these serious flaws with the SFA-CHD hypothesis were emerging, other evidence also emerged indicating that refined carbohydrates, sugar, whole grains, and dietary fiber play important roles in CHD.

Comparisons have been made between the impact of carbohydrates and of SFA on the risk of CHD. A meta-analysis of cohort studies reported that a 5% lower intake of SFA combined with a 5% higher intake of carbohydrates has little impact on risk of CHD [36]. The hazard ratio (HR) was 1.07 (CI 1.01–1.14) for coronary events, and 0.96 (CI 0.82, 1.13) for coronary deaths. These observations are consistent with those from experimental studies. Replacing dietary SFA with carbohydrates lowers the TC, the LDL-cholesterol, and the HDL-cholesterol resulting in a negligible change in the ratio of TC to HDL-cholesterol [37]. This would be predicted to have no impact on risk of CHD [14]. However, replacing dietary SFA with carbohydrates does cause a rise in the blood level of triglycerides [37] and this may cause some elevation in risk [38]. Unfortunately, these findings are difficult to interpret as the carbohydrates in these studies are likely to come from mixed sources including sugar, refined cereals, and whole grains. However, as the large majority of carbohydrates in diets in North America and Europe come from refined carbohydrates, the above findings are mainly a reflection of the action of refined carbohydrates. We can reasonably conclude, therefore, that when these studies indicate that “carbohydrates” have a very similar effect on risk of CHD as does SFA (i.e., an increase in risk of a few percent), it is refined carbohydrates that are largely responsible.

There is an almost complete absence of evidence from RCTs regarding the effect of replacing dietary fat with carbohydrates on the incidence of CHD. The Women’s Health Initiative was conducted in the USA on 49,000 postmenopausal women [39]. The experimental diet was reduced in total fat from 36% to 29% of energy while carbohydrates were increased. Both SFA and PUFA were reduced. There was little change in blood lipids. No reduction in risk of CHD was seen after 8 years of follow up. This failure to lower the risk of CHD is consistent with the lack of change in blood lipids.

Especially insightful findings came from two cohort studies: the Nurses’ Health Study and the Health Professionals Follow-up Study. The combined results showed that intake of carbohydrates from refined starches and added sugars was positively associated with risk of CHD (HR: 1.10, CI 1.00–1.21, based on comparison of extreme quintiles) [40]. The results also revealed a protective association for intake of carbohydrates from whole grains (HR: 0.90, CI 0.83 to 0.98). It was estimated that replacing 5% of energy intake from SFA with equivalent energy intake from carbohydrates from whole grains was associated with 9% lower risk of CHD (HR: 0.91, CI 0.85 to 0.98) while replacing SFA with carbohydrates from refined starches/added sugars was not significantly associated with CHD.

Cereal fiber has served as the commonly used proxy measure for whole grains in most cohort studies. A meta-analysis of cohort studies reported that cereal fiber has a protective association with risk of CHD [24]. The pooled estimate for the risk ratio per 7 g/day increase in fiber from cereal sources was 0.84 (CI 0.76–0.94). A similar association was seen for insoluble fiber. Data from the Nurses’ Health Study II, a cohort study of American women, observed that the median intakes of cereals in the extreme quintiles were 3.1 and 8.8 g/day [41]. The difference (5.7 g/day) will be somewhat more in men. Thus, 7 g/day of cereal fiber, which is the amount supplied by roughly 62 g of whole grain cereal, is the approximate difference between the extreme quintiles of intake in Americans. Thus, this amount of whole grain cereal may reduce risk of CHD by approximately 16%.

The colon has an enormous content of microbiota, now commonly referred to as the microbiome. Findings in recent years have linked dysbiosis (an imbalance in the microbiome) with various diseases [42,43], including cardiovascular disease [44,45]. Several possible mechanisms have been suggested by which abnormal functioning of the microbiome may play a role in the etiology of cardiovascular disease [44]. Dietary fiber affects the microbiome [46] and this may be a mechanism by which cereal fiber is protective against CHD.

Many studies have been made concerning the relationship between sugar, sugar-sweetened beverages (SSBs), and CHD. A meta-analysis of cohort studies reported that intake of SSBs is associated with risk of CHD [47]. A 22% greater risk of myocardial infarction was seen for each additional daily serving (RR: 1.22, CI 1.14–1.30). These findings are consistent with the results of RCTs which reveal that sugar causes an increase in the blood pressure and the blood level of triglycerides, TC, and LDL-cholesterol [48].

In summary, replacing dietary intake of SFA with refined starches has little effect on the risk of CHD. However, consumption of added sugars, especially of SSBs, may have a stronger association with risk than either SFA or refined starches. When SFA are replaced with whole grains, risk of CHD is decreased. However, there is still uncertainty regarding the absolute and relative importance of these different components of the diet. A growing weight of authoritative opinion is emerging that supports these conclusions [29,30,31].

## 6. Future Research Studies

A notable lesson from the story told here is that carrying out research into the relationship between diet and CHD is very challenging. This is best exemplified by the Women’s Health Initiative (Howard et al.), which was discussed earlier [39]. The study was commenced in the early 1990s. The focus of this RCT was entirely on the *quantity* of dietary fats and carbohydrates but not on their *quality.* It is now clear that the study design was seriously flawed which resulted in a failure to achieve any impact on risk of CHD.

The two types of research study that have consistently yielded our most valuable information are RCTs and cohort studies. Evidence coming from RCTs is usually placed above evidence from cohort studies in the research hierarchy. However, Temple [49] argued that in the area of diet and health findings from RCTs are not necessarily more reliable than those from well-designed cohort studies. Of paramount importance is to be constantly aware that both research strategies are prone to various types of error. RCTs often use subjects at relatively high risk of the disease being investigated. This may limit whether the findings are valid for healthy persons. RCTs often have a relatively short period of follow up which again may limit the validity of the findings. Cohort studies have their own sources of error. A potential source of significant error may occur in the measurement of habitual food intake. Confounding due to such factors as socioeconomic status may also introduce significant error. In order to minimize this problem the results from cohort studies are normally analyzed by multivariate analysis but residual confounding can still occur.

It must be stressed that many variables come into play when conducting research in the area of diet and health, including CHD. These include components of the diet and attributes of the subjects (such as age, ethnicity, and health). Another important variable is the substances in the blood that may be related to the development of CHD. For example, a recent development is the recognition of lipoprotein subclasses [50,51,52]. Related to this is the use of metabolomics for the identification of diverse substances associated with CHD [53]. This line of investigation may lead to the identification of new biomarkers that can serve as valuable tools in CHD research.

## 7. Summary

The key points made in this review are summarized in Table 1. It is now clear that the role of SFA in the causation of CHD has been much exaggerated. Until the 1980s the weight of evidence indicated that a high intake of SFA causes a raised TC which may then lead to CHD. But evidence that has steadily accumulated since around 1990 demonstrates that SFA plays a relatively minor role in CHD. The findings from many cohort studies have provided strong evidence that SFA has a much weaker association with risk of CHD than do several other dietary factors including trans fats (which increase risk), as well as fish, and fruit and vegetables (which reduce risk). The belief that a reduced intake of SFA is the key dietary change for the prevention of CHD led millions of people to increase their intake of carbohydrates, most of which were refined. But this dietary change has a negligible effect on the ratio of TC to HDL-cholesterol and, in consequence, has no impact on risk of CHD. A reduced intake of SFA combined with an increased intake of PUFA may reduce the risk of CHD, but even that assertion has been questioned.

Growing evidence points to refined carbohydrates, especially SSBs, as being linked to risk of CHD. Conversely, whole grains are protective. An extra one or 2 servings per day of these foods increases risk (in the case of refined carbohydrates/SSBs) or decreases risk (in the case of whole grains) by approximately 10% to 20%, which is similar to the strength of the associations seen for trans fats, fish, and fruit and vegetables. Most cohort studies have measured intake of cereal fiber rather than whole grains. It is probable that whole grains contain many substances, in addition to fiber, that exert a protective action against CHD.

## Figures and Tables

**Table 1:** Relationship between fat, sugar, whole grains, and coronary heart disease: A summary.

Claimed Relationship between Diet and CHD	Time Period	Supporting Evidence
Refined carbohydrates, sugar, and a low intake of dietary fiber	Late 1960s and through the 1970s	International comparisons of diet and incidence of CHD
A high intake of SFA is a major cause of CHD.	Roughly 1974 to 2014	The TC level is a major risk factor for CHD. SFA increases the TC. Partially replacing SFA with PUFA lowers the TC and reduces risk for CHD.
The role of SFA in the causation of CHD has been much exaggerated. A high intake of SFA is a fairly minor cause of CHD.	Starting around 2014	Cohort studies show that intake of SFA has only a weak association with risk of CHD. The strongest indicator of risk of CHD is the ratio of TC to HDL-cholesterol. Based on changes in this ratio it is predicted that partially replacing dietary SFA with PUFA reduces the risk of CHD whereas replacing dietary SFA with refined carbohydrates does not reduce the risk of CHD.
Refined starches/added sugars/sugar-sweetened beverages are an important cause of CHD. Cereal fiber/whole grains reduce risk.	Starting around 2000	Cohort studies. Sugar causes an increase in risk factors for CHD (blood pressure and blood lipids).

HD-coronary heart disease; PUFA-polyunsaturated fatty acids; RCTs-randomized controlled trials; SFA-saturated fatty acids; TC-total cholesterol (blood cholesterol level).

## References

[1] Yudkin J. (1972). Pure, White and Deadly.

[2] Yudkin J. (1972). Sucrose and cardiovascular disease. Proc. Nutr. Soc..

[3] CBC Sugar Industry Paid Scientists for Favourable Research, Documents Reveal. http://www.cbc.ca/news/health/sugar-harvard-conspiracy-1.3759582.

[4] Cleave T.K., Campbell G.D. (1966). Diabetes, Coronary Thrombosis and the Saccharine Disease.

[5] Cleave T.K. (1974). The Saccharine Disease.

[6] Burkitt D.P., Trowell H.C. (1975). Refined Carbohydrate Foods and Disease: Some Implications of Dietary Fibre.

[7] Trowell H. (1972). Ischemic heart disease and dietary fiber. Am. J. Clin. Nutr..

[8] Walker A.R. (1951). Cereals, phytic acid and calcification. Lancet.

[9] McRorie J.W., McKeown N.M. (2017). Understanding the physics of functional fibers in the gastrointestinal tract: An evidence-based approach to resolving enduring misconceptions about insoluble and soluble fiber. J. Acad. Nutr. Diet..

[10] Kahn H.A., Dawber T.R. (1966). The development of coronary heart disease in relation to sequential biennial measures of cholesterol in the Framingham study. J. Chronic Dis..

[11] Keys A., Anderson J.T., Grande F. (1957). Prediction of serum-cholesterol responses of man to changes in fats in the diet. Lancet.

[12] McGandy R.B., Hegsted D.M., Myers M.L., Stare F.J. (1966). Dietary carbohydrate and serum cholesterol levels in man. Am. J. Clin. Nutr..

[13] Keys A. (1980). Seven Countries: A Multivariate Analysis of Death and Coronary Heart Disease.

[14] Mozaffarian D., Micha R., Wallace S. (2010). Effects on coronary heart disease of increasing polyunsaturated fat in place of saturated fat: A systematic review and meta-analysis of randomized controlled trials. PLoS Med..

[15] Ravnskov U. (1998). The questionable role of saturated and polyunsaturated fatty acids in cardiovascular disease. J. Clin. Epidemiol..

[16] Temple N.J. (1983). Coronary heart disease—Dietary lipids or refined carbohydrates?. Med. Hypotheses.

[17] Jakobsen M.U., Overvad K., Dyerberg J., Schroll M., Heitmann B.L. (2004). Dietary fat and risk of coronary heart disease: Possible effect modification by gender and age. Am. J. Epidemiol..

[18] Leosdottir M., Nilsson P.M., Nilsson J.A., Berglund G. (2007). Cardiovascular event risk in relation to dietary fat intake in middle-aged individuals: Data from the Malmo diet and cancer study. Eur. J. Cardiovasc. Prev. Rehabil..

[19] McGee D., Reed D., Stemmerman G., Rhoads G., Yano K., Feinleib M. (1985). The relationship of dietary fat and cholesterol to mortality in 10 years: The Honolulu heart program. Int. J. Epidemiol..

[20] Oh K., Hu F.B., Manson J.E., Stampfer M.J., Willett W.C. (2005). Dietary fat intake and risk of coronary heart disease in women: 20 years of follow-up of the nurses’ health study. Am. J. Epidemiol..

[21] Posner B.M., Cobb J.L., Belanger A.J., Cupples L.A., D’Agostino R.B., Stokes J. (1991). Dietary lipid predictors of coronary heart disease in men. The Framingham study. Arch. Intern. Med..

[22] De Souza R.J., Mente A., Maroleanu A., Cozma A.I., Ha V., Kishibe T., Uleryk E., Budylowski P., Schünemann H., Beyene J. (2015). Intake of saturated and trans unsaturated fatty acids and risk of all cause mortality, cardiovascular disease, and type 2 diabetes: Systematic review and meta-analysis of observational studies. BMJ.

[23] Zheng J., Huang T., Yu Y., Hu X., Yang B., Li D. (2012). Fish consumption and CHD mortality: An updated meta-analysis of seventeen cohort studies. Public Health Nutr..

[24] Threapleton D.E., Greenwood D.C., Evans C.E., Cleghorn C.L., Nykjaer C., Woodhead C., Cade J.E., Gale C.P., Burley V.J. (2013). Dietary fiber intake and risk of cardiovascular disease: Systematic review and meta-analysis. BMJ.

[25] Gan Y., Tong X., Li L., Cao S., Yin X., Gao C., Herath C., Li W., Jin Z., Chen Y. (2015). Consumption of fruit and vegetable and risk of coronary heart disease: A meta-analysis of prospective cohort studies. Int. J. Cardiol..

[26] Gordon T., Castelli W.P., Hjortland M.C., Kannel W.B., Dawber T.R. (1977). High density lipoprotein as a protective factor against coronary heart disease. The Framingham study. Am. J. Med..

[27] Lewington S., Whitlock G., Clarke R., Sherliker P., Emberson J., Halsey J., Qizilbash N., Peto R., Collins R., Prospective Studies Collaboration (2007). Blood cholesterol and vascular mortality by age, sex, and blood pressure: A meta-analysis of individual data from 61 prospective studies with 55,000 vascular deaths. Lancet.

[28] Farvid M.S., Ding M., Pan A., Sun Q., Chiuve S.E., Steffen L.M., Willett W.C., Hu F.B. (2014). Dietary linoleic acid and risk of coronary heart disease: A systematic review and meta-analysis of prospective cohort studies. Circulation.

[29] Sacks F.M., Lichtenstein A.H., Wu J.H.Y., Appel L.J., Creager M.A., Kris-Etherton P.M., Miller M., Rimm E.B., Rudel L.L., Robinson J.G. (2017). Dietary fats and cardiovascular disease: A presidential advisory from the American Heart Association. Circulation.

[30] Liu A.G., Ford N.A., Hu F.B., Zelman K.M., Mozaffarian D., Kris-Etherton P.M. (2017). A healthy approach to dietary fats: Understanding the science and taking action to reduce consumer confusion. Nutr. J..

[31] Zock P.L., Blom W.A., Nettleton J.A., Hornstra G. (2016). Progressing insights into the role of dietary fats in the prevention of cardiovascular disease. Curr. Cardiol. Rep..

[32] Siri-Tarino P.W., Chiu S., Bergeron N., Krauss R.M. (2015). Saturated fats versus polyunsaturated fats versus carbohydrates for cardiovascular disease prevention and treatment. Annu. Rev. Nutr..

[33] Hamley S. (2017). The effect of replacing saturated fat with mostly *n*-6 polyunsaturated fat on coronary heart disease: A meta-analysis of randomised controlled trials. Nutr. J..

[34] Ramsden C.E., Zamora D., Majchrzak-Hong S., Faurot K.R., Broste S.K., Frantz R.P., Davis J.M., Ringel A., Suchindran C.M., Hibbeln J.R. (2016). Re-evaluation of the traditional diet-heart hypothesis: Analysis of recovered data from Minnesota coronary experiment (1968–73). BMJ.

[35] Mozaffarian D. (2016). Dietary and policy priorities for cardiovascular disease, diabetes, and obesity: A comprehensive review. Circulation.

[36] Jakobsen M.U., O’Reilly E.J., Heitmann B.L., Pereira M.A., Bälter K., Fraser G.E., Goldbourt U., Hallmans G., Knekt P., Liu S. (2009). Major types of dietary fat and risk of coronary heart disease: A pooled analysis of 11 cohort studies. Am. J. Clin. Nutr..

[37] Mensink R.P., Zock P.L., Kester A.D., Katan M.B. (2003). Effects of dietary fatty acids and carbohydrates on the ratio of serum total to HDL cholesterol and on serum lipids and apolipoproteins: A meta-analysis of 60 controlled trials. Am. J. Clin. Nutr..

[38] Cullen P. (2000). Evidence that triglycerides are an independent coronary heart disease risk factor. Am. J. Cardiol..

[39] Howard B.V., Van Horn L., Hsia J., Manson J.E., Stefanick M.L., Wassertheil-Smoller S., Kuller L.H., LaCroix A.Z., Langer R.D., Lasser N.L. (2006). Low-fat dietary pattern and risk of cardiovascular disease: The women’s health initiative randomized controlled dietary modification trial. JAMA.

[40] Li Y., Hruby A., Bernstein A.M., Ley S.H., Wang D.D., Chiuve S.E., Sampson L., Rexrode K.M., Rimm E.B., Willett W.C. (2015). Saturated fats compared with unsaturated fats and sources of carbohydrates in relation to risk of coronary heart disease: A prospective cohort study. J. Am. Coll. Cardiol..

[41] Schulze M.B., Liu S., Rimm E.B., Manson J.E., Willett W.C., Hu F.B. (2004). Glycemic index, glycemic load, and dietary fiber intake and incidence of type 2 diabetes in younger and middle-aged women. Am. J. Clin. Nutr..

[42] Lynch S.V., Pedersen O. (2016). The human intestinal microbiome in health and disease. N. Engl. J. Med..

[43] Robles Alonso V., Guarner F. (2013). Linking the gut microbiota to human health. Br. J. Nutr..

[44] Ahmadmehrabi S., Tang W.H.W. (2017). Gut microbiome and its role in cardiovascular diseases. Curr. Opin. Cardiol..

[45] Ettinger G., MacDonald K., Reid G., Burton J.P. (2014). The influence of the human microbiome and probiotics on cardiovascular health. Gut Microbes.

[46] Flint H.J., Duncan S.H., Scott K.P., Louis P. (2007). Interactions and competition within the microbial community of the human colon: Links between diet and health. Environ. Microbiol..

[47] Narain A., Kwok C.S., Mamas M.A. (2016). Soft drinks and sweetened beverages and the risk of cardiovascular disease and mortality: A systematic review and meta-analysis. Int. J. Clin. Pract..

[48] Te Morenga L.A., Howatson A.J., Jones R.M., Mann J. (2014). Dietary sugars and cardiometabolic risk: Systematic review and meta-analyses of randomized controlled trials of the effects on blood pressure and lipids. Am. J. Clin. Nutr..

[49] Temple N.J. (2016). How reliable are randomised controlled trials for studying the relationship between diet and disease? A narrative review. Br. J. Nutr..

[50] Sarzynski M.A., Burton J., Rankinen T., Blair S.N., Church T.S., Després J.P., Hagberg J.M., Landers-Ramos R., Leon A.S., Mikus C.R. (2015). The effects of exercise on the lipoprotein subclass profile: A meta-analysis of 10 interventions. Atherosclerosis.

[51] Phillips C.M., Perry I.J. (2015). Lipoprotein particle subclass profiles among metabolically healthy and unhealthy obese and non-obese adults: Does size matter?. Atherosclerosis.

[52] Tian L., Li C., Liu Y., Chen Y., Fu M. (2014). The value and distribution of high-density lipoprotein subclass in patients with acute coronary syndrome. PLoS ONE..

[53] Li Y., Zhang D., He Y., Chen C., Song C., Zhao Y., Bai Y., Wang Y., Pu J., Chen J. (2017). Investigation of novel metabolites potentially involved in the pathogenesis of coronary heart disease using a UHPLC-QTOF/MS-based metabolomics approach. Sci. Rep..

